# Immunometabolism at the Nexus of Cancer Therapeutic Efficacy and Resistance

**DOI:** 10.3389/fimmu.2021.657293

**Published:** 2021-05-17

**Authors:** Javier Traba, Michael N. Sack, Thomas A. Waldmann, Olga M. Anton

**Affiliations:** ^1^ Departamento de Biología Molecular, Centro de Biología Molecular Severo Ochoa, Consejo Superior de Investigaciones Científicas-Universidad Autónoma de Madrid (CSIC-UAM), Madrid, Spain; ^2^ Cardiovascular Branch, National Heart, Lung and Blood Institute, NIH, Bethesda, MD, United States; ^3^ Lymphoid Malignancies Branch, Center for Cancer Research, National Cancer Institute, NIH, Bethesda, MD, United States

**Keywords:** immunometabolism, cancer, immune cells, T cells, NK cells, macrophages, immunosuppression, metabolites

## Abstract

Constitutive activity of the immune surveillance system detects and kills cancerous cells, although many cancers have developed strategies to avoid detection and to resist their destruction. Cancer immunotherapy entails the manipulation of components of the endogenous immune system as targeted approaches to control and destroy cancer cells. Since one of the major limitations for the antitumor activity of immune cells is the immunosuppressive tumor microenvironment (TME), boosting the immune system to overcome the inhibition provided by the TME is a critical component of oncotherapeutics. In this article, we discuss the main effects of the TME on the metabolism and function of immune cells, and review emerging strategies to potentiate immune cell metabolism to promote antitumor effects either as monotherapeutics or in combination with conventional chemotherapy to optimize cancer management.

## Introduction

Cancer is a highly heterogeneous disease that constitutes a major worldwide health problem. Cancer cell presents the following main hallmarks: self-sufficiency in growth signals, insensitivity to anti-growth signals, evading apoptosis, limitless replicative potential, sustained angiogenesis, reprogramming of energy metabolism, evading immune destruction and tissue invasion and metastasis (1). An additional and important characteristic of cancer cells is that they recruit a repertoire of healthy cells that contribute to tumorigenesis, such as fibroblasts (the predominant cell type), pericytes, endothelial cells, mesenchymal stem cells, macrophages and lymphocytes (2). This review deals with two of those important hallmarks: reprogramming of energy metabolism and evasion of immune destruction.

Quiescent cells in the human body typically display a catabolic metabolism that mainly relies on oxidative phosphorylation, utilizing glucose, fatty acids or amino acids as substrates. In contrast, proliferating cells, such as rapidly dividing immune cells, switch to an anabolic metabolism, rely on glycolysis for energy generation and utilize large amounts of glucose and glutamine as building blocks for lipids, nucleic acids and amino acids for proliferation. Immunometabolism is an emerging concept that studies metabolic changes that occur in immune cells to support their functions. These changes are critical for the appropriate immune response, as they control downstream transcriptional and posttranscriptional events, and their dysregulation may compromise growth, proliferation and effector functions. As a general overview, in an inflammatory context immune cells will upregulate aerobic glycolysis (such is the case in B cells, effector Th1 and Th17 T cells, M1 macrophages, dendritic cells and Natural Killer cells), while a metabolism relying more on the oxidative phosphorylation usually supports an anti-inflammatory phenotype (as is the case in M2 macrophages and regulatory T (T_reg_) cells).

Cancer cells also undergo changes in metabolism that are required to support their bioenergetic needs and biosynthesis requirements for fast growth and invasiveness, and in fact, metabolic reprogramming is now considered one of the hallmarks of the cancer cell (3). Metabolic changes in pro-inflammatory immune and cancer cells mirror each other, which leads to competition for nutrients and oxygen in the tumor microenvironment (TME). In addition, cancer cells and other cells embedded in the tumor secrete metabolites and inhibitory cytokines that interfere with the targeting of immune cells to eliminate tumor cells. These characteristics represent the way tumoral cells have evolved to create a favorable environment for their growth and development while evading and suppressing the immune response. The aim of immunotherapy is to modulate the host immune system to attack cancer cells with tools such as immune checkpoint blockade, T cell therapy or cancer vaccines, and it has been shown to be effective (4). The alteration of metabolic pathways involved in cancer-induced immune failure seem to be a suitable target to enhance existing immunotherapies.

## Overview of Cellular Metabolism and Cancer Cell Metabolism

Cells utilize a number of substrates, including glucose, fatty acids and amino acids (such as glutamine), to obtain energy in the form of adenosine triphosphate (ATP), precursors for biosynthesis and redox equivalents. A key pathway is glycolysis, which consists on the catabolism of glucose to generate pyruvate and 2 molecules of ATP. Pyruvate can in turn be transformed into lactate (anaerobic glycolysis) or preferentially (under normoxic conditions) transferred to the mitochondria where it is transformed into acetyl coenzyme A (acetyl-CoA), which is then metabolized by the tricarboxylic acid (TCA) cycle to produce ATP, CO_2_ and reduced nicotinamide adenine dinucleotide (NADH). NADH is oxidized by the electron transport chain (ETC) of the inner mitochondrial membrane, which transfers its electrons sequentially *via* redox reactions to several respiratory complexes (I, III and IV), and finally to O_2_ as final acceptor. Electron transport is an exergonic process and is coupled to the pumping of protons (H^+^) by those complexes to the intermembrane space, generating an electrochemical gradient that is used by the ATP synthase to generate ATP, a process called oxidative phosphorylation (OxPhos). Cells can also burn fatty acids in the mitochondria in a process called fatty acid β-oxidation (FAO). This process yields large amounts of acetyl-CoA and redox equivalents that can be oxidized by the ETC.

**Figure 1:**
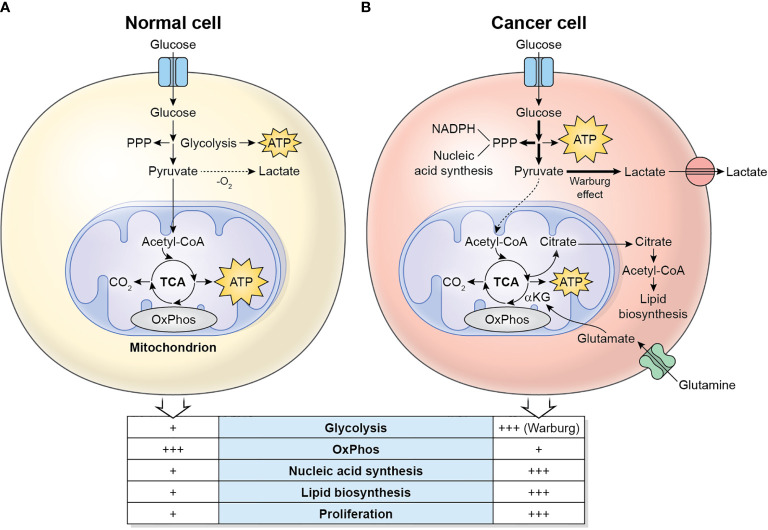
Metabolic differences between normal cells **(A)** and cancer cells **(B)**. Normal/quiescent cells in aerobiosis metabolize glucose mainly to pyruvate, which is then oxidized in the mitochondria to CO_2_ using the TCA cycle and OxPhos for the generation of ATP. ATP synthesis takes places preferentially in the mitochondria. In anaerobiosis, pyruvate is metabolized to lactate instead. Cancer cells in aerobiosis or anaerobiosis convert most glucose to lactate (which is exported to the TME), while diverting some glycolytic intermediates to the PPP, which generates NADPH and pentoses for the synthesis of nucleic acids. ATP synthesis is largely cytosolic. Glutaminolysis generates glutamate, which is converted to α-ketoglutarate, a major substrate to refuel the TCA cycle. The TCA cycle intermediate citrate is exported to the cytosol, where it is converted to acetyl-CoA, used for the synthesis of lipids.

The main metabolic differences between normal cells and cancer cells are described in **Figure 1**. Cancer cells display a metabolic switch that supports their rapid proliferation. In these cells, even in the presence of oxygen, pyruvate is not used by the TCA cycle, but instead converted to lactate by lactate dehydrogenase A (LDHA). This phenomenon is called Warburg effect or aerobic glycolysis (5). The excess lactate produced by the proliferating cancer cells is exported by monocarboxylate transporters (MCTs) and increases the acidity of the TME. Cancer cells also divert significant amounts of glycolytic intermediates into the pentose phosphate pathway (PPP) to generate NADPH (used for reductive anabolism and to reduce the disulfide form of glutathione, GSSG, to the sulfhydryl form, GSH, which is an antioxidant) and pentoses, including ribose-5-phosphate (for nucleic acid synthesis). Glutamine is also a major nutrient in cancer cells, which in fact display glutamine addiction, and is utilized as a source of nitrogen for biosynthetic pathways, including the synthesis of non-essential amino acids and nucleotides (by participating in nitrogen-donating reactions), and to replenish the TCA cycle (anaplerosis) which is used to synthesize large amounts of lipids from citrate (6). Glutamine is initially converted to glutamate by the enzyme Glutaminase, and then to alpha-ketoglutarate, by Glutamate Dehydrogenase (GDH), which fuels the TCA cycle. Cancer cells also display a lipogenic phenotype, showing increased *de novo* synthesis of fatty acids. Remarkably, the highly active metabolic activity of the tumor cell depletes nutrients and O_2_ in the TME and renders it hostile to immune cells.

Cellular metabolism has been proved a key factor to regulate cellular responses. In the field of immune cells, immunometabolism has become a new target to control and modulate immune responses, with special relevance in the fields of immunotherapy and cancer.

## Overview of Immunometabolism

**Figure 2:**
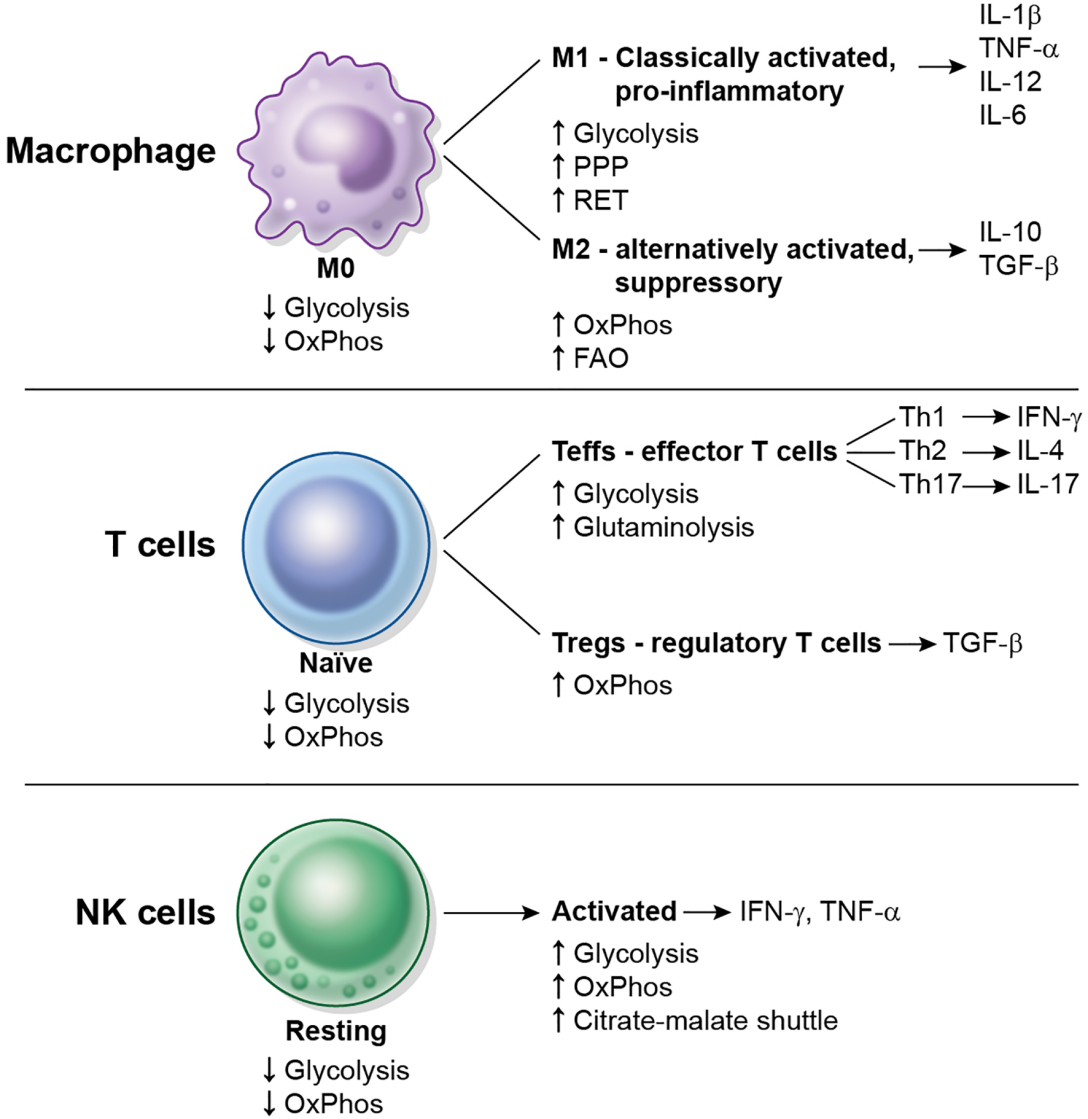
Overview of metabolic changes in some immune cell types upon activation. Macrophages, CD4^+^ T cells and NK cells are shown. Resting or naïve cells typically display a low metabolic rate (low glycolysis, low OxPhos). M0 macrophages differentiate into M1 and M2 macrophages, naïve CD4+ T cells differentiate into Teffs or Tregs, and resting NK cells become activated. Each cell subtype displays different metabolic requirements and repertoire of cytokines to function.

Most resting immune cells are relatively metabolically inactive. The concept of immunometabolism illustrates the changes in metabolism and special needs that immune cells undergo to be able to fulfill their specific functions. Immunometabolism has been most extensively studied in the modulation of macrophage polarization and of CD4^+^ T cell activation. The metabolic changes that occur in macrophages, CD4^+^ T cells and Natural Killer (NK) cells after activation are schematized in **Figure 2**. The characterization of immunometabolism is being extended to other immune cells such as dendritic cells (7, 8), neutrophils, myeloid-derived suppressor cells (MDSC), innate lymphoid cells (ILC) (9), B cells (10) and plasma cells (11).

### Immunometabolism of Macrophages

Macrophages are a multifunctional type of leukocyte that plays a role in innate immunity, but also collaborate in the initiation of adaptive immunity, as they are antigen presenting cells (APCs) to helper T cells. They are terminally differentiated cells and perform functions related to tissue homeostasis, repair, phagocytosis and development.

There are distinct varieties of macrophages in several tissues, including Kupffer cells, alveolar macrophages, osteoclasts, peritoneal macrophages, microglia, and others. Macrophages either derive from circulating monocytes or are established in tissues before birth and then maintained during adult life independently of monocytes (12).

After activation using *in vitro* stimuli, macrophages can be broadly divided into two subgroups: M1, classically activated or proinflammatory macrophages, generated *in vitro* by incubation with bacterial-derived products such as lipopolysaccharide (LPS) that bind to Toll-like receptors (TLRs), and signals associated with infection such as interferon γ (IFN-γ); and M2, alternatively activated or reparative macrophages, generated *in vitro* by incubation with interleukin (IL)-4, IL-10 or IL-13. It is probable that macrophage polarity *in vivo* is much more complex and cells display a spectrum of functional phenotypes between M1 and M2 subtypes.

In solid tumors there is a population of macrophages called tumor-associated macrophages (TAMs) that typically resembles M2 macrophages and exert immunosuppressive and pro-tumorigenic functions (13), including stimulation of angiogenesis, which contributes to nutritional support of the tumor, and remodeling of the extracellular matrix. TAMs constitute the largest population of myeloid cells that infiltrate solid tumors.

#### M1 Macrophages

M1 macrophage secrete proinflammatory cytokines such as IL-1β, tumor necrosis factor (TNF-α), IL-6 or IL-12 that initiate the immune response, perform phagocytosis of microbes and generate reactive oxygen species (ROS), and metabolize arginine using inducible nitric oxide synthase (iNOS) to generate nitric oxide (NO).

##### Early Metabolic Changes in M1 Activation

During early activation of the M1 phenotype, the Toll-like receptor 4 (TLR4) agonist LPS induces a rapid induction in both glycolysis and mitochondrial oxygen consumption, as well as an increases in glucose uptake and the levels of TCA cycle metabolic intermediates (14). Metabolites typical of M1 macrophages such as lactate, itaconate or succinate do not reach high levels until after 24 hours. Glucose-derived pyruvate is taken up by the mitochondria and oxidized. Increased citrate is exported to the cytosol through the mitochondrial citrate carrier (CIC) and converted to acetyl-CoA by the ATP-citrate lyase enzyme (ACLY), which is then utilized not only for fatty acid and cholesterol synthesis, but also for acetylation reactions. Thus, LPS stimulates glucose-dependent histone acetylation and pro-inflammatory gene expression at early time points after M1 activation.

##### Late Changes in M1 Activation

The late metabolism of the M1 cell is mainly glycolytic, and they also display increased fatty acid synthesis and increased flux though the PPP, involved in the synthesis of NADPH which is in turn required for fatty acid synthesis, ROS generation (by NADPH oxidase), and NO synthesis. The TCA cycle becomes incomplete, as two breaks occur (15), which leads to the accumulation of specific metabolites: the first break is caused by a decrease in the expression of isocitrate dehydrogenase (IDH), and leads to citrate accumulation, which is required for lipids, prostaglandins and itaconate synthesis. Itaconic acid is formed from cis-aconitate by the inducible Immune-Responsive Gene 1 (IRG1), and is an anti-microbial compound. Because of the first brake in the cycle, alpha-ketoglutarate in M1 macrophages is derived from glutamine rather than from glucose. The second break occurs at succinate dehydrogenase, with accumulation of succinate, required for hypoxia-inducible factor 1 alpha (HIF-1α) stabilization, a key transcription factor for pro-inflammatory genes such as IL-1β (16). Because of the second break in the cycle, malate in M1 cells is thus generated *via* the arginosuccinate shunt. The increased generation of ATP by glycolysis in the cytosol of M1 cells decreases significantly the requirement for mitochondrial OxPhos to supply ATP to the cell. As a consequence, M1 cells do not synthesize significant amounts of ATP in the mitochondria, and perform instead reverse electron transport (RET), which is the flow of electrons from ubiquinol back to respiratory complex I for the generation of ROS in the mitochondrial matrix (17, 18). These electrons typically come from succinate oxidation by respiratory complex II, succinate dehydrogenase.

Other late metabolic changes in M1 cells include: increased expression of acetyl-CoA carboxylase (ACC), which leads to increased levels of malonyl-Coenzyme A in the cytosol and increased protein malonylation (19). One of the substrates of this modification is the glycolytic enzyme glyceraldehyde 3-phosphate dehydrogenase (GAPDH), which converts glyceraldehyde 3-phosphate into 1,3-bisphosphoglycerate. GAPDH binds to the 3’ UTR region of the mRNA of TNF-α and blocks its translation. After its malonylation, GAPDH detaches from the mRNA and allows the synthesis of TNF-α protein. M1 differentiation also leads to an increase in succinylation of proteins (16), possibly because of elevated succinate levels, although its consequences have not been explored in detail.

M1 polarization is irreversible (20), as subsequent incubation of M1 cells with IL-4 or IL-10 does not turn the cells into M2. The main reason for this is that the mitochondrial respiratory chain is permanently damaged by NO after M1 differentiation, and accordingly, inducible NO synthase (iNOS) inhibition allows M1 cells to repolarize to M2.

##### Regulation of Cytokine Production by Glycolysis

In macrophages and monocytes glucose availability controls the expression of proinflammatory cytokines by several mechanisms. The first involves regulation of mRNA translation: in situations of low glycolysis the glycolytic enzyme GAPDH binds, as explained above, to the TNF-α mRNAs and blocks its translation (21). However, during active glycolysis GAPDH detaches from the mRNA which allows high production of the cytokine. A second mechanisms deal with the transcription of the mRNA for IL-1β. After activation of the macrophage, glycolysis drives an increase in the levels of succinate (derived from glutamine) (16), which in turn control the stability of HIF-1α. Under basal conditions, HIF-1α is hydroxylated by prolyl-hydroxylases (PDHs) and targeted for degradation by the proteasome. However, under limited oxygen or under high ROS levels, the activity of PHD is impaired and this leads to HIF-1α accumulation. The model that emerges from these studies is that glycolytic ATP drives M1 macrophage activation by providing an increase in mitochondrial membrane potential that is absolutely required for succinate oxidation-dependent RET to occur, leading to a large increase in mitochondrial ROS levels that facilitate HIF-1α accumulation, which binds directly to the promoter of the IL1B gene and favors expression of pro-inflammatory genes (17). This process reciprocally decreases anti-inflammatory cytokines, such as IL-10.

#### M2 Macrophages

Alternatively-activated macrophages do not secrete pro-inflammatory cytokines and are involved in tissue recovery, remodeling of the extracellular matrix or immunosupression. Another signature of these cells is highly N-glycosylated lectin and mannose receptors and thus there is an increase in the metabolism of nucleotide sugars such as uridine diphosphate N-acetylglucosamine (UDP-GlcNAc), UDP-glucose, and UDP-glucuronate (15). They are also characterized by utilization of arginine *via* Arginase 1 (Arg1), which catalyzes its conversion to L-ornithine, and by the secretion of TFG-β and IL-10.

In contrast to M1 cells, M2 cells use OxPhos (including FAO) to support ATP synthesis. Strikingly, high glycolysis is also required for M2 polarization, since its blockade blunts expression of M2 markers such as Arg1 or resistin-like molecule alpha (Retnla) (22, 23), which suggests that glycolysis probably provides pyruvate for the TCA cycle, and recent studies suggest that FAO is largely not essential for M2 polarization (23, 24). Knockdown of pyruvate dehydrogenase kinase 1 (PDK1), an enzyme that drives inhibitory phosphorylation on the pyruvate dehydrogenase complex, increases conversion of glucose-derived pyruvate to acetyl-CoA and enhances M2 differentiation while preventing M1 differentiation (23).

IL-4 addition to macrophages increased glucose uptake and oxygen consumption, and this depended on Akt and mTOR1, which regulate ACLY phosphorylation to control cytosolic/nuclear acetyl-CoA levels for acetylation of histones on some M2 genes (22). IL-4 also induces phosphorylation and activation of the transcription factor signal transducer and activator of transcription 6 (STAT6), which induces expression of protein peroxisome proliferator-activated receptor gamma (PPARγ) coactivator-1β (PGC-1β), which in turn stimulates mitochondrial biogenesis and OxPhos (25). PGC-1β also coactivates STAT6-responsive genes, such as Arg1. Interestingly, overexpression of PGC-1β strongly stimulates FAO and potentiates M2 differentiation without altering STAT6 phosphorylation, while it prevents M1 differentiation (25). Similarly to PGC-1β, PPARγ is required for M2 activation, mitochondrial biogenesis and stimulation of fatty acid metabolism as well (26).

### Immunometabolism of T Cells

T cells are leukocytes involved in adaptive immunity. T cells are largely divided into two subtypes: T helper (Th) cells, also known as CD4^+^ cells, that participate in the activation of other immune cell types (B cells, cytotoxic cells and macrophages) by the release of cytokines; and T cytotoxic (Tc) cells, also known as CD8^+^ cells or cytotoxic T lymphocytes (CTLs), that kill cancer and virus-infected cells.

#### CD4^+^ T Cells

Naïve CD4^+^ T cells have a very low metabolic rate that depends mainly on OxPhos using pyruvate and FAO. Stimulated CD4^+^ T cells can differentiate into effector T cells (Teff), including T helper 1 (Th1), T helper 2 (Th2) and T helper 17 (Th17) subsets, which switch to an anabolic state that is orchestrated by mTOR, and inducible regulatory T cells (Treg), which do not depend on mTOR, but on 5’ adenosine monophosphate-activated protein kinase (AMPK). Th1 cells are involved in Type 1 or cell-mediated immunity, which includes neutrophils, natural killer cells, CD8^+^ cytotoxic T cells and classically activated macrophages (M1), and responds to intracellular pathogens, bacteria and viruses (27). Th2 cells are involved in Type 2 immunity, which includes eosinophils, mast cells, basophils and alternatively activated macrophages (M2), and typically regulates tissue repair and regeneration, but also responds to extracellular parasites and helminths (28). Th17 are involved in Type 3 immunity, which responds to extracellular bacteria and fungi, and are characterized by the capacity to release IL-17 (and IL-22). Other minor subsets of T helper cells include T helper 9 (Th9) cells, which secrete IL-9. Type 1 and 3 immunity largely mediate autoimmune diseases, with Th17 cells playing an important role in the pathogenesis of diseases such as psoriasis, rheumatoid arthritis or asthma (27), whereas type 2 immunity can cause allergic diseases. All 3 types of immune responses display substantial cross−regulation.

##### Effector T Cells

Upon *in vitro* activation of the T cell receptor (TCR) with anti-CD3 and -CD28 antibodies, there is a dramatic increase in glycolysis and OxPhos in order to support their proliferation and biosynthesis, and the oxygen consumption/glycolysis ratio greatly decreases. Fatty acid and pyruvate oxidation decrease compared to resting T cells, while the increase in OxPhos is likely fueled by glutamine oxidation in the TCA cycle (29). Interestingly, OxPhos, but not glycolysis, is required during the initial activation of the cell that is accompanied by cell growth but not proliferation (29, 30). However, once the T cell has been activated for at least 48 hours, either glycolysis or OxPhos are enough to sustain survival or proliferation (30), while glycolysis remains essential for some effector functions (see below).

The increase in glycolysis is mainly driven by an increase in the glucose transporter GLUT1 and the glycolytic enzymes hexokinase 2 (HK2), pyruvate kinase M2 (PKM2) and LDHA (29), while the increase in OxPhos comes from a large activation of mitochondrial biogenesis, with increasing levels of mitochondrial DNA and mitochondrial proteins (31). Interestingly, while there is a gradual increase in GLUT1 along several days after activation, mitochondrial biogenesis largely occurs during the first 24 hours (31). There is a concomitant elevation in ROS levels, which is important as a second signal (32). Additionally, the expression of glutaminolysis-associated genes such as glutaminase 2 (GLS2) is upregulated, including transporters of glutamine and amino acids, and there is an increase in the flux though the PPP, involved in the biosynthesis of ribose-5-phosphate for nucleic acids and NADPH, required for lipid synthesis. This extensive metabolic remodeling requires mTOR and NO (31), and is accompanied by upregulation of transcription factors and signaling pathways, including the proto-oncogene Myc that regulates and supports the anabolic needs of proliferating T cells. Deletion of Myc prevents activation-induced increases in glycolysis and glutaminolysis in T cells (29). Th17 cells also utilize the transcription factor HIF-1α, which binds to the promoter of retinoid-related orphan receptor-γ (RORγt), a lineage-specific marker of Th17 cells (33).

The energy sensor 5’ adenosine monophosphate-activated protein kinase (AMPK), which is activated by an increased ratio of AMP to ATP, is required for metabolic adaptation and flexibility under conditions of limited nutrient availability. Indeed, energy depletion by reduced glucose concentration or nutrient limitation is sensed by AMPK, which is phosphorylated in threonine 172 of its alpha subunit, and drives the expression of genes involved in glutamine metabolism such as glutamine transporters or glutaminase (34), which fuel mitochondrial metabolism under these conditions. This leads to a decrease in glycolysis, while oxygen consumption and cellular ATP levels are largely maintained.

##### Regulation of Cytokine Production by Glycolysis

T cell activation leads to an increase in IFN-γ production by at least three mechanisms: the first mechanism is epigenetic and involves increased expression LDHA by Myc and HIF-1α, which promotes aerobic glycolysis and generation of ATP in the cytosol. As a result, in the presence of glucose, citrate is not required by the TCA for mitochondrial ATP generation and can rather be exported to the cytosol and converted to acetyl-CoA by ACLY, where it is used as a substrate by histone acetyltransferases for regulation of gene expression, including IFN-γ gene transcription (35). The second mechanism is similar to the regulation of TNF-α expression in macrophages and involves regulation of mRNA translation: in situations of low glycolysis the glycolytic enzyme GAPDH binds to the 3’ UTR regions of IL-2 and IFN-γ mRNAs and blocks their translation. On the other hand, during active glycolysis GAPDH detaches from mRNAs which allows high production of cytokines (30). AMPK seems to be involved in this translational mechanism of regulation as well: AMPK activators such as 5-aminoimidazole-4-carboxamide ribonucleotide (AICAR) decrease IFN-γ production (34), possibly by inhibition of mTOR. AMPKα-1 deficiency leads to increase production of IFN-γ, which becomes resistant to AICAR inhibition (34), and to increased translation of the IFN-γ mRNA. The third mechanism involves regulation of Ca^2+^ signaling by the glycolytic metabolite phosphoenolpyruvate (PEP), which accumulates in activated T cells, as they express the M2 isoform of pyruvate kinase (PKM2) (29, 36) which is preferentially a dimer and enzymatically less active in the conversion of PEP to pyruvate than the tetrameric PKM1 isoform. PEP accumulates in the presence of glucose and inhibits the sarco/endoplasmic reticulum Ca^2+^ (SERCA)-ATPase (a P-type ATPase whose function is to pump and sequester Ca^2+^ into the endoplasmic reticulum), thus increasing cytosolic Ca^2+^ transients after TCR signaling and activation of the nuclear factor of activated T cells (NFAT) transcription factor (37). Accordingly, glucose depletion in T cells prevents accumulation of PEP after TCR signaling, and active SERCA limits cytosolic Ca^2+^ transients, which leads to cytoplasmatic localization of NFAT and decreased expression of IFN-γ.

##### Regulatory T Cells

In contrast to effector cells, Tregs express low levels of GLUT1, utilize mainly FAO and depend on the expression of the transcription factor Forkhead box P3 (Foxp3). Tregs do not use mTOR or HIF-1α, but constitutively show high levels of phosphorylated AMPK, which positively regulates FAO and blocks mTORC1 activity.

Consistent with their dependence on FAO rather than glycolysis, the deletion of HIF-1α promotes rather than inhibits Treg differentiation, likely due to the ability of HIF-1α to bind to and degrade Foxp3. Rather, Tregs shows high levels of phosphorylated AMP-activated protein kinase (AMPK), which regulates FAO by phosphorylating and inhibiting acetyl-CoA carboxylase (ACC) thereby activating carnitine palmitoyl transferase 1a (CPT1a), the rate-limiting step in FAO (38).

Interestingly, glycolysis and mTORC1 are essential to support the migration of Tregs (38). Tumor-infiltrating Tregs, which may constitute up to 20–30% of the total CD4^+^ population of the tumor (39), are strongly associated with advanced cancer stage and poor prognosis.

#### CD8^+^ T Cells

Naïve CD8^+^ T cells have a similar metabolism to naïve CD4^+^ T cells. Stimulated CD8^+^ T cells differentiate into effector cells. The best characterized effector CD8^+^ T cell subpopulation are Tc1 cells, which are promoted by IL-12 and IFN-γ, secrete cytokines such as IFN-γ and TNF-α and have high cytolytic potential against cells infected with intracellular pathogens by releasing cytotoxic molecules, such as granzymes and perforin. In contrast, Tc2 cells secrete IL-4, IL-5 and IL-13, but not IFN-γ, and display reduced cytotoxic activity compared to Tc1 cells. Other subsets of effector CD8^+^ T cells include Tc9 and Tc17. Activation of CD8^+^ T, as in CD4^+^ T cells, also induces cell growth and proliferation, and a metabolic switch to aerobic glycolysis that depends on Glut1, mTOR and Myc.

During resolution of an immune response, surviving T cells convert to memory T cells. Interestingly, while effector CD8^+^ T cells are highly glycolytic, CD8^+^ T memory cells rely on OxPhos again. OxPhos and FAO are essential for these cells to respond upon re-exposure to the antigen and for longevity.

### Immunometabolism of NK Cells

NK cells are cytotoxic lymphocytes involved in anti-tumor and anti-viral innate immunity. NK cells do not express polymorphic germline-encoded receptors, such as TCR or BCR, nor require prior sensitization (40), and the activation of their cytolytic functions is prompted by the engagement of receptors that recognize invariable ligands on the surface of a target cell (41, 42). The balance of the signal coming from activating and inhibitory receptors will dictate the activating or inhibitory fate of the NK cell response. If they get activated, NK cells will kill target cells by releasing lytic granules (which contain perforin and granzyme) or activating cell death receptors on their targets. NK cell activation response also induces secretion of cytokines, such as IFNγ or TNFα.

Resting NK cells have very low basal metabolic rates and utilize OxPhos primarily. While short times of cytokine stimulation or receptor signaling does not alter metabolic parameters significantly (43), overnight incubations with IL-2 or IL-15 induce an increase in OxPhos and especially glycolysis (44, 45). Activated NK cells metabolize pyruvate into acetyl-CoA and then engage the citrate–malate shuttle, which consists of the export of citrate (generated by condensation of acetyl-CoA and oxaloacetate by citrate synthase) into the cytosol *via* CIC in exchange for malate, its breakdown by Acly into acetyl-CoA and oxaloacetate, which is metabolized by malate dehydrogenase 1 (MDH1), consuming cytosolic NADH and yielding cytosolic malate that is exchanged for citrate though CIC, and then oxidized in the mitochondria to oxaloacetate by malate dehydrogenase 2 (MDH2) with the generation of NADH. Thus, this process bypasses the TCA cycle, and generates both mitochondrial NADH to support OxPhos and cytosolic acetyl-CoA to support acetylation reactions and fatty acid synthesis (46). NK cell activation is also associated with increased mitochondrial mass (46) and increased mitochondrial membrane potential (47). Interestingly, unlike in other types of lymphocytes, glutamine is not metabolized through glutaminolysis or the TCA cycle in activated NK cells (48).

There are different pathologies that can alter NK cells metabolism, including cancer (49), obesity (50) and viral infections (51). As many other immune cells, NK cell metabolism regulation depends on mTORC1 (52). Upon cytokine stimulation, mTORC1 activity increases and enhances glycolysis rate. mTORC1 inhibitors, such as rapamycin, results in inhibition of glycolysis but not OxPhos (52, 53). mTORC2 is closely related to mTORC1, and they share the kinase core subunit, but differ in some specific subunits (which makes mTORC2 insensitive to inhibition with rapamycin). mTORC2 inhibits mTORC1 activity, which results in an inhibition of NK cell functions (54).

Other regulators of NK cells metabolism are transcriptional factor cMyc, controlled by the availability of glutamine and other amino acids (55), and the sterol regulatory element binding protein (SREBP), driven by mTORC1 signaling, essential for glycolysis and OxPhos regulation (46).

### Fibroblasts

Fibroblasts are not immune cells, but cells that have a role in synthesizing extracellular matrix proteins. They are however the predominant cell type in some tumors and contribute to tumorigenesis. Interestingly, transplantation of tumor cells together with cancer-associated fibroblast (CAFs) leads to more malignant cancers compared to tumor cells alone or with normal fibroblasts. From the immunometabolic perspective, oxygen depletion in the TME promotes, *via* epigenetic reprogramming, a metabolic switch in CAFs leading to a glycolytic metabolism that fuels biosynthetic pathways of cancer cells, including the PPP and nucleic acid metabolism (56, 57). Metabolites secreted by CAFs and taken up by tumors include lactate (58), glutamine (59, 60) and lipids (61), among others. CAFs also promote the recruitment of monocytes towards a tumor, their differentiation into macrophages, and their polarization to an M2 phenotype (62, 63). Interestingly, there is reciprocal crosstalk between CAFs and TAMs (64), as TAMs are able to activate normal fibroblasts into CAFs (62).

## Tumor Microenvironment (TME) and Metabolic Reprograming of Immune Cells

**Figure 3:**
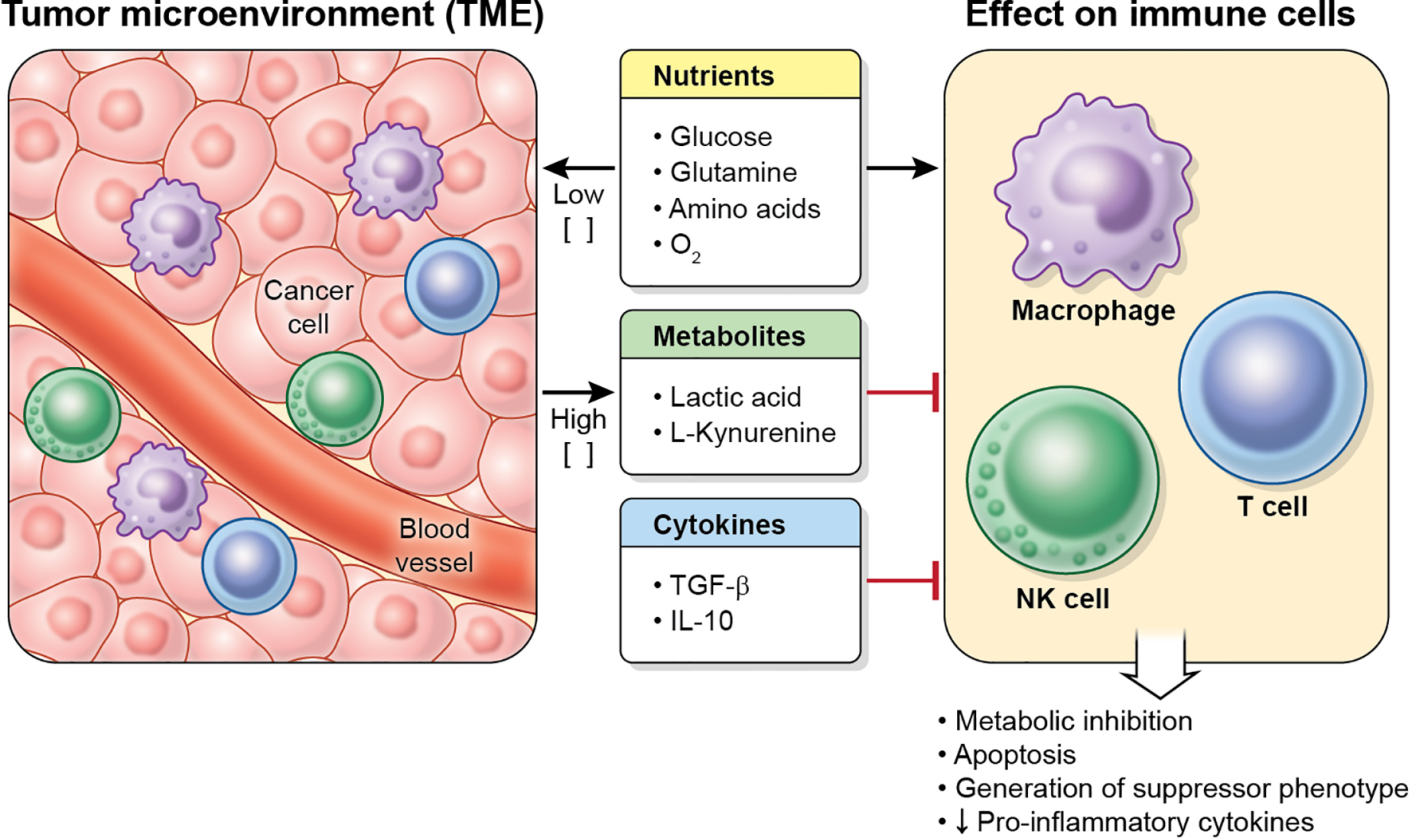
Characteristics of the TME. Tumor cells (and other cells in the tumor) deplete nutrient levels (glucose, glutamine, amino acids, O_2_, etc.) in the TME, increase the levels of some metabolites, such as lactic acid or L-kynurenine, and secrete cytokines (including TGF-β and IL-10), which inhibit the metabolism, effector function and proliferation in immune cell, and may cause apoptosis.

The cancer metabolic phenotype generates a milieu that is hostile to proinflammatory immune cells. This TME exerts immunosuppressive effects in immune cells by several mechanisms, including competition for nutrients, and secretion of metabolites and cytokines (**Figure 3**). In addition, the TME may alter dysfunctional immune cells to make them transition into tumor-supporting cells. These effects on macrophages, T cells and NK cells are reviewed below.

### Metabolic Competition for Nutrients

Tumor cells are known to be highly glycolytic and solid tumors consume large amounts of glucose and other nutrients, which results in low extracellular levels of glucose, glutamine and other amino acids, including arginine and tryptophan (65), and this blunts antitumor responses through multiple mechanisms.

A mouse sarcoma model shows that tumor-mediated glucose depletion in the TME skews the differentiation of macrophages toward the M2 phenotype (66). Unlike M1 macrophages, M2 macrophages do not compete for glucose with the tumor cells, as they preferentially employ OxPhos.

Intratumoral CD4^+^ T cells in Braf/Pten melanoma-bearing mice express a glucose-deprivation transcriptional signature, suggesting that they experience glucose deprivation *in vivo* and that their glycolysis is indeed restricted (37). Although glucose depletion does not lead to defects in proliferation or survival in T cells, it leads to decreased synthesis of cytokines, suppresses activation and may limit antitumor responses. Inhibition of glycolysis also decreases the expression of perforin and granzymes, and blocks cytolytic activity in CD8^+^ cells (67). Indeed, it has been shown in a mouse sarcoma model that tumor-mediated glucose depletion in the TME inhibits mTOR activity and glycolysis in CD8^+^ T cells and dampens their ability to produce cytokines (66). Glutamine depletion leads to defects in T cell proliferation and synthesis of IL-2 and IFN-γ (68).

In NK cells, limited glucose directly reduces glycolysis and OxPhos, but will also affect the activity of regulators of the activation such as mTORC1 (44). Consequently, glucose depletion significantly impairs IFN-γ production (69), proliferation and cytotoxicity (51). NK cells express several glucose transporters (GLUT1, GLUT3 and GLUT4) and it has been shown that cytokine stimulation increases expression of GLUT1, needed to increase the uptake of glucose essential to fuel the augmented glycolysis rate that will support the activating functions of NK cells. Interestingly, glutaminolysis does not sustain OxPhos in activated NK cells, that rely on the citrate–malate shuttle instead of a full TCA cycle, but supports cMyc expression (48). Accordingly, glutamine depletion leads to a rapid loss of c-myc protein and loss of effector functions.

#### Hypoxia

Due to their high metabolic profile and sometimes poor vascularization, tumors also contain areas of hypoxia.

During hypoxia, NK cells upregulate HIF-1α, but lose their ability to upregulate the surface expression activating receptors in response to IL‐2 or IL‐15, including NKp46, NKp30, NKp44, and NKG2D, as well as their capacity to kill target cells (70). It has also been shown that hypoxia modifies the NK cell transcriptome and reduces release of some cytokines including IFN-γ and TNF-α in response to several stimuli or soluble cytokines, although it sustains the expression of chemokine receptors, including CCR7 and CXCR4 (71).

TAMs in oxygen-rich regions of the tumor, such as those close to blood vessels, display features of M1 macrophages, whereas TAMs in hypoxic regions of the tumor display M2-like features (72). Hypoxia stimulates the expression of HIF-1α in TAMs, which controls the levels of proteins involved in angiogenesis, such as vascular endothelial growth factor (VEGF), or metastasis, such as regulated in development and DNA damage response 1 (REDD1) (72, 73). Interestingly, REDD1 is an mTOR inhibitor, which decreases glycolysis in the hypoxic TAM, leading to more glucose available for endothelial cells and an excessive angiogenic response, which in turn leads to aberrant and dysfunctional blood vessel formation and increased metastasis. When REDD1 is depleted, an mTOR-mediated glycolytic enhancement in the TAM leads to glucose competition with blood vessels and their normalization (73).

### Metabolites Secreted by Tumor Cells

#### Lactic Acid

Due to their glycolytic metabolism, tumor cells secrete large amounts of lactic acid that can reach up to 30–40 mM, which generates regions of high acidity in the TME. In fact, a negative correlation between tumor LDHA expression and patient survival has been found in melanoma (74). In CD8^+^ T cells lactic acid, but not its sodium salt, inhibits the expression of IFN-γ and IL-2, suppresses proliferation and cytotoxic activity, and may induced cell death (74, 75).

In NK cells, lactic acid also inhibits the expression of IFN-γ and induces apoptosis in a mitochondrial ROS-dependent fashion (74, 76, 77). Interestingly, lactate suppresses IFN-γ expression even at physiologic pH in NK cells (77).

Lactate also inhibits the release of proinflammatory cytokines in macrophages (78) and induces M2 macrophage polarization (79). Conditioned medium from lactate-treated macrophages stimulates angiogenesis, and proliferation and migration of cancer cells *in vitro (* 79). Lactic acid induces expression of VEGF, Arg1 and other M2 genes in TAMs *via* HIF-1α stabilization, and this is critical for tumor growth, as Arg1-deficient macrophages impair tumor progression *in vivo* in a mouse model (80).

#### l-kynurenine

l-kynurenine, a tryptophan-derived catabolite resulting from the activity of indoleamine 2,3-dioxygenase (IDO), an enzyme that is often expressed in tumor cells or tumor-associated cells (TAMs, dendritic cells, etc.) (81, 82), interferes with NK cells by regulating the surface expression of activating receptors and thus their antitumor function (83). In additional studies, IDO is also shown to inhibit NK cell proliferation *via* l-kynurenine (84, 85).

l-kynurenine also inhibits proliferation of CD4^+^ and CD8^+^ T effector cells (86) and induces the generation of Tregs by interacting with the aryl hydrocarbon receptor (AHR) (87). Furthermore, a blockade of IDO leads to conversion of Tregs to T_h_17 cells (88), which highlights the role of IDO in the choice between immunosuppression and immune activation. Importantly, increased IDO expression correlates with poor prognosis in some cancers (89). IDO causes inhibition of effector T cells also by an additional mechanisms: it leads to tryptophan depletion in the TME, which activates the protein general control nonderepressible 2 (GCN2) in T cells, a stress-response kinase that is activated by elevations in uncharged tRNA. GCN2 function is to phosphorylate eukaryotic Initiation Factor 2 alpha (eIF2α) to activate the integrated stress response, which leads to a decrease in global protein synthesis and the induction of selected genes, which leads to CD8^+^ T cell anergy (90). Strikingly, in a mouse model of skin carcinogenesis, the absence of GCN2 does not phenocopy the absence of IDO, which promotes resistance against tumor development, suggesting the existence of additional pathways that operate downstream of IDO (91). This additional pathway is mTOR, which senses tryptophan depletion.

### Cytokines Present in the TME

#### TGF-β

TGF-β inhibits cytokine-induced metabolic changes and effector functions in NK cells, including expression of CD71 (transferrin receptor), granzyme B, IFN-γ and the stimulatory receptor CD69, *via* mTORC1-dependent and independent pathways (92, 93). It has been suggested that upregulation of the gluconeogenic enzyme Fructose-1,6-bisphosphatase 1 (FBP1) in NK cells of lung cancer, which inhibits their glycolytic metabolism, is caused by high levels of TGF-β in the TME (94). TGF-β also supports the induction of Tregs in tumors (95).

#### IL-10

This cytokine is frequently upregulated in cancers and has been shown to directly affect the function of APCs by inhibiting the expression of major histocompatibility complex (MHC) class II and costimulatory molecules CD80/B7-1 and CD86/B7.2, which in turn induces immune suppression or tolerance (96). The role IL-10 in macrophage immunometabolism is well stablished. This cytokine opposes the switch to the M1 metabolic program in macrophages by inducing mitochondrial oxygen consumption and increasing mitochondrial integrity *via* mTOR, by decreasing mitochondrial ROS levels, by decreasing the cell surface expression of GLUT1 and thus by inhibiting glycolysis (97).

On the other hand, IL-10 downregulates the expression of Th1 cytokines (TNF-α, IFN-γ), inhibits CD4^+^ and CD8^+^ T cell proliferation and production of IFN-γ and IL-2 (98), and induces T-regulatory responses. IL-10 deficiency also impairs Treg function and enhances Th1 and Th17 immunity to inhibit tumor growth (99).

Finally, IL-10 suppresses production of IFN-γ by NK cells without altering cytotoxicity (100).

## Targeting Immunometabolism for Immunotherapy

Immunotherapy involves the use of the immune system as therapy to treat cancer. Current therapies include blockade of immune checkpoint receptors (which maintain self-tolerance and prevent uncontrolled inflammation) and adoptive cell transfer. However, these techniques have their limitations, including T and NK cell exhaustion, possibly because of the effects of the TME. This section describes some immunotherapies in T cells, NK cells and macrophages and briefly describes some strategies used to modulate the metabolism of the tumor cell or the immune cell that can be combined with other therapies to boost the immune response to cancer.

### Targeting T Cells

T cells have been successfully used for immunotherapy in a number of ways, including immune checkpoint blockade, which uses monoclonal antibodies to bind and inactivate inhibitory receptors such as cytotoxic T-lymphocyte-associated protein 4 (CTLA-4) and programmed cell death-1 (PD-1) or its ligand programmed cell death ligand 1 (PD-L1) (101), or adoptive cell transfer, which involves the isolation of T cells, improving their anti-tumor capacity *in vitro*, expanding them in culture, and transferring them back to the patient (102). Adoptive cell transfer was improved by generation of T cells that express engineered TCRs that target a specific antigen and lately by the generation of chimeric antigen receptor (CAR) T cells, which express an artificial chimeric antigen-binding receptor that combines both antigen-binding and T-cell activating functions into a single molecule (103). Some strategies to indirectly or directly target the metabolism of T cells and improve immunotherapy are described here.

Pharmacological inhibition of the lactate transporters MCT1 and MCT4 using diclofenac restores T cell function, slows tumor growth and improves the efficacy of checkpoint inhibition therapies (104). Knockdown of LDHA in tumor cells using RNAi nanoparticles neutralized the pH of the TME, increased infiltration with CD8^+^ T and NK cells, decreased the number of Tregs, significantly inhibited the growth of tumors and potentiated checkpoint inhibition therapy (105).

Removal of extracellular kynurenine in the TME by administration of bacterial kynureninase linked to polyethylene glycol (for prolonged systemic retention) increases the frequency of effector CD8^+^ T cells in the tumor (but not in the periphery), reduces tumor growth, increases survival in a CD8^+^ cell-dependent manner and potentiates checkpoint inhibition therapy (106). In a mouse model of melanoma, the combination of the tryptophan analog 1-methyl-D-tryptophan plus an antitumor vaccine caused conversion of Tregs to the Th17, with marked enhancement of CD8^+^ T cell activation and antitumor efficacy (88). It is interesting to remark that the IDO inhibitor 1-methyl-tryptophan exists in two stereoisomers, and it has been shown that the L isomer (1-methyl-L-tryptophan) is the more potent inhibitor of IDO activity. Remarkably, the D isomer (Indoximod), although it does not bind to, or biochemically inhibit IDO, nor prevents the production of kynurenine, is much more effective in reversing the suppression of T cells and has stronger therapeutic effects (107). This probably happens *via* direct restoration of mTOR activity, which is suppressed due to tryptophan deprivation (91, 108). Several small-molecule IDO inhibitors, including Indoximod, Navoximod, Epacadostat or BMS-986205, are currently being used in several clinical trials (108)

JHU083, a pro-drug of 6-diazo-5-oxo-L-norleucine (DON), a glutamine antagonist which inhibits glutaminase and other glutamine-requiring enzymes, impairs tumor cell metabolism, which in turn increases the availability of nutrients and oxygen in the TME. Remarkably, in the presence of JHU083, tumor infiltrating CD8^+^ T cells display increased glycolysis and mitochondrial oxygen consumption and regain their effector functions and proliferation. This is probably because of the plasticity of T cells, which in the absence of glutamine metabolism undergo a metabolic reprogram and are able to replenish the TCA using glucose anaplerosis *via* pyruvate carboxylase and acetate metabolism, while tumor cells do not possess that plasticity (109).

Overexpression of phosphoenolpyruvate carboxykinase 1 (PCK1), which converts oxaloacetate into PEP and CO_2_, increases PEP levels in T cells even in glucose depleted conditions and consequently restores cytosolic Ca^2+^ transients, nuclear localization of NFAT and IFN-γ expression (37). Furthermore, after adoptive transfer of PCK1-overexpressing CD4^+^ or CD8^+^ T cells into a melanoma-bearing mice, there was a decrease in tumor growth which prolonged survival of the mice (37).

### Targeting NK Cells

NK cells are also being used for immune checkpoint blockade therapies: blocking of NKG2A, an inhibitory receptor, by the use of antibodies improves NK-mediated cytotoxicity and this is currently being used in ongoing clinical trials (110). Adoptive cell transfer with NK cells is also a promising therapy: NK cells may be treated with IL-15, other cytokines or drugs prior to their transfer back to the patient (111). Antibody blockade of PD-1 or CTLA-4 in combination with IL-15 has also been used (112) Some strategies to target the metabolism of NK cells and improve immunotherapy are described here.

Adoptive transfer of NK cells treated with MB05032, an FBP1 inhibitor that restores NK cell glycolysis and effector functions, slows tumor growth in a lung cancer model in mouse (94).

In mice, systemic pH buffering of the tissue milieu by supplementing drinking water with sodium bicarbonate normalizes IFN-γ expression but not cytotoxic capacity in NK cells, and delays tumor growth (77).

Blockade of glutaminase should not affect NK cells effector functions, since they do not metabolize glutamine to glutamate, but use it for the import of other amino acids in order to sustain mTOR function and cMyc expression (48). Since MYC in NK cells is degraded by glycogen synthase kinase 3 (GSK3), inhibitors of this enzyme should also activate effector functions in these cells. Indeed, increased antitumor activity in NK cells has been shown after *ex vivo* pharmacologic inhibition of GSK with CHIR99021 or other compounds followed by adoptive transfer (113, 114), although it appears that GSK3 inhibition leads to activation of the NF-αB pathway and increased expression of TNF-α, and only to a modest increase in IFN-γ, and whether the metabolism of the NK cells or MYC levels were altered was not explored in those studies.

### Targeting Macrophages

Therapies that deal with macrophages mainly consists of two approaches, those that deplete TAMs in an effort to prevent their tumor supporting functions (chemokine (C-C motif) ligand 2 (CCL2) or CC‐chemokine receptor 2 (CCR2) blockade, which prevents their recruitment into tumors, for example), and those that try to repolarize them towards an M1-like antitumor phenotype (115). Some strategies to target the metabolism of macrophages and improve immunotherapy are described here.

Pharmacologic or genetic blockade of glutamine synthetase, the enzyme that converts glutamate into glutamine, alters the metabolism of macrophages and skews their differentiation from the M2 phenotype to M1 in an HIF-1α-dependent fashion, which leads to decreased T cell suppression, decreased angiogenesis and prevention of metastasis in a Lewis lung carcinoma mouse model (116).

TAMs express high levels of the T cell checkpoint receptor PD1, and these PD1-containing TAMs are preferentially M2 macrophages (117). Strikingly, the frequency of M2 PD-1 containing TAMs increased with disease stage in human cancer patients. PD-1 expression in TAMs correlates negatively with phagocytic potency against tumor cells, and blockade of PD-1 or PD-L1 *in vivo* increases macrophage phagocytosis, reduces tumor growth and increases the survival of mice in mouse models of cancer in a macrophage-dependent fashion, as depletion of TAMs abrogates these effects (117). Remarkably, TAMs (and even mouse bone marrow-derived macrophages or human monocyte-derived macrophages) express PD-L1 as well (118). Blockade of PD-L1 with antibodies induces macrophage activation, release of proinflammatory cytokines and an activation of mTOR, suggesting that PD-L1 constitutively provides a negative signal to these macrophages (118). In addition, blockade of PD-L1 in macrophages alters the transcriptome to resemble that of M1 proinflammatory cells (118). In a mouse model of melanoma, *in vivo* treatment of RAG^-/-^ mice (which lack functional T cells) with PD-L1 antibodies triggers TAM activation to a proinflammatory state and a significant slowing of tumor growth which is independent of T cells, the main target for immune checkpoint blockers (118).

Respiratory complex I inhibitor metformin (an anti-diabetic drug) inhibits M2 polarization of macrophages and blocks the migration-promoting and angiogenesis-promoting properties of conditioned media from M2 cells (119). Metformin also inhibits metastasis in a Lewis lung carcinoma model, and this effect is abolished when TAMs are depleted, which suggests that metformin indeed targets macrophages in this model (119).

### Nutrient Restriction as Therapy

Because of a strong competition at the TME, it might be argued that dietary interventions that limit nutrient availability in an attempt to prevent the growth of cancer cells may also deprive immune cells of those very same nutrients, thus raising concerns about the possible detrimental effect of suppressing anti-cancer immunity. Despite this, fasting, long-term fasting, fasting-mimicking diets, and ketogenic diets are currently under investigation in trials for several cancer types. Fasting has been shown not only to be useful as therapy against cancer by several mechanisms, but also to increase tolerance to chemotherapy, with reduction of many side effects (120), as it protects normal cells, but not cancer cells, from high doses of chemotherapy by a mechanism that involves a reduction in the levels of circulating insulin-like growth factor-I (121, 122).

From the immunometabolic perspective, fasting suppresses the polarization of TAMs towards the M2 phenotype, possibly because of decreased presence of adenosine in the TME (123), a metabolite that directly restricts antitumor responses by activating the adenosine A2A receptor (A2AR) in T-cells (124). Low-protein diet also shifts the polarization of TAMs toward the inflammatory M1 phenotype and increases the response to immunotherapies (125). Nutrient restriction is also able to increase mitochondrial ROS, including superoxide levels, in several cancer cell types, which increases the efficacy of chemotherapy (126, 127). However, since fasting and caloric restriction are hard to implement in the clinical setting, especially in cancer patients that are struggling with cachexia or loss of appetite, the use of caloric restriction mimetics such as resveratrol or anti-hyperglycemic agents such as metformin, which might provide the same effect without the need to fast, is increasingly being used in the field (128). In fact, the caloric restriction mimetic hydroxycitrate improves antitumor immunity by depleting Tregs in the tumor, and enhances immunotherapy (129).

## Other Factors That Influence Cancer Immunometabolism and Immunotherapy

### Obesity

Obesity is a disease characterized by the excessive accumulation of fat tissue and lipids along the body. The accumulation of lipids occurs inside immune cells as well, reprograming their metabolism and affecting their function. Obesity is characterized by the activation of mTOR signaling because of nutrients excess. The contribution of obesity to cancer is two-fold: it drives a low-grade sistemic chronic inflammatory state but it also blocks cancer immune surveillance by metabolically reprogramming immune cells and affecting their function (130).

In NK cells, obesity downregulates the transcription of genes involved in NK-cell mediated cytotoxicity, resulting in an impairment of killing function and less production of IFN- γ. NK cells from obese individuals display an impaired ability to increase glycolysis and OXPHOS after stimulation with cytokines, and this effect depends on PPARα/δ (50). The number of circulating NK cells is reduced in obese people compared to control individuals and lipid treated NK cells fail to control tumor growth *in vivo*.

In the case of T cells, lipid accumulation supports Treg function, which relies on FAO to fuel OxPhos (131). On the other hand, the chronic inflammatory environment present in obesity suppresses Teff, which show an exhausted phenotype with impaired cytokine secretion (132, 133). Consistent with this, high fat diet in mice accelerates tumor growth, and the mechanism involves depletion of intratumoral T cells and an increase in MDSCs and TAMs (134). Interestingly, tumor cells and T cells adapt differently to lipid accumulation and undergo opposing metabolic changes: while tumor cells increase fatty acid utilization, CD8^+^ T cells do not (134). The increase in TAMs is consistent with lipid accumulation, since FAO is associated with the M2 suppressor phenotype (25, 135).

Regarding immunotherapy, and taking into account the multiple suppressor effects of obesity in the immune system, it could be assumed that obesity might contribute negatively to the efficacy of immunotherapies, but this is not always the case. In obese individuals there is an increased expression of PD-1, an inhibitory checkpoint molecule, likely due to increased levels of leptin, an hormone produced by adipose tissue. High expression of PD-1 has been generally linked to T-cell exhaustion, but it also means that therapies targeting PD-1 might have more efficacy (136, 137). When PD-1 is upregulated in T cells, the interaction with its ligand PD-L1 (on tumor cells) will inhibit the anti-tumor T-cell activity. Anti PD-1-PD-1L therapy will block this interaction and allow T cells to better fulfill their tumor killing function. Some studies show and association between obesity and immune checkpoint blockade immunotherapy, being this therapy more successful in patients with higher expression of PD-L1 (138).

### Sex-Related Differences

There are fundamental metabolic aspects that are different between males and females. For instance, sex differences are apparent in adipose tissue distribution and, as discussed previously, fat tissue and lipids may influence immune cells and metabolism. Also, there are sex differences in immune responses: males are more prone to developing certain infections and cancer types whereas females experience higher risk to developing autoimmune diseases (139). Factors that could potentially contribute to these differences are steroid hormones, sex chromosomes (overall the genes that escape to X-chromosome inactivation), mitochondrial function and environmental factors (139).

As adult females display stronger innate and adaptive immune responses than males, they present increased susceptibility to inflammatory and autoimmune diseases, but lower risk for cancer. Interestingly, neutrophils in females display an activated neutrophil profile characterized by an upregulation of the pathway for type I interferons, enhanced proinflammatory responses, and distinct bioenergetics, which are driven by the sex hormone estradiol (140). We have not covered neutrophils in this review, but there is growing evidence that suggests an important role of this leukocytes, especially tumor-associated neutrophils (TANs), in cancer progression (141). There is currently a debate and conflicting results on whether there are differences in the efficacy of immunotherapy between men and women (142), and this is probably because of sampling bias in clinical trials.

### Aging

Age is a risk factor a number of pathologies. It is associated with metabolic disease, autoimmunity, higher risk of infections, and cancer. There are plenty of metabolic changes associated with aging: a decline in mitochondrial activity (143), decreases in NAD levels (144) and low grade chronic inflammation (145).

Aging is a strong risk factor for cancer. In fact, subcutaneously injected tumors grow faster in old mice compared to young mice (146). It is known that Treg cells accumulate in spleens and lymph nodes of old mice, and depletion of those cells induces a strong cytotoxic response and protects old mice against some tumors (147). M2 macrophages are also increased in tissues of old mice, and these macrophages appear to be hypersensitive to tumor-derived stimuli, as they secrete higher amounts of immunosuppressive cytokines, including IL-4 and TGF-β (148). Interestingly, several immunotherapies are less effective in aged animals, while others such as immune checkpoint blockade remain useful (149). Older human adults are under-represented in clinical trials in general, although they seem to benefit similarly to younger patients from checkpoint blockade (150).

## Concluding Remarks

Immunotherapy is becoming increasingly successful for many cancers, especially against hematological malignancies. However, immunotherapy still fails for a substantial numbers of patients, especially in those with solid tumors, and one of the reasons for that failure is that the TME limits the killing capacity of these cells and makes the patients refractory to such therapy. Strategies to bypass the inhibitory effect of the TME in proinflammatory cells are currently being developed. They are key for the success of immunotherapies, and the study of immunometabolism is thus a critical field for cancer research. Targeting immunometabolism during immunotherapy is proving to be challenging, as tumor cells and inflammatory immune cells utilize similar metabolic pathways, and thus targeting a given pathway, such as tumor glucose metabolism to reverse the Warburg phenotype by use of glycolysis inhibitors, may concurrently disrupt the tumor lytic effect of immune cells. Subtle differences between the cancer cell and the immune cell and or TME and/or immune cell targeted metabolic modulation strategies should be investigated as alternate strategies to enhance the cancer disease spectrum for immunotherapy.

## Author Contributions

JT and OA conceived the work. JT, MS, TW and OA wrote the manuscript. All authors contributed to the article and approved the submitted version.

## Funding

This work was supported by the Intramural Research Programs of the National Institutes of Health, National Cancer Institute and National Heart, Lung, and Blood Institute. JT is supported by the Ministry of Science and Innovation (MICINN) of Spain (grants RYC2018-026050-I and PID2019-105665RA-I00).

## Conflict of Interest

The authors declare that the research was conducted in the absence of any commercial or financial relationships that could be construed as a potential conflict of interest.
